# Effect of Mesos Components (MgSO_4_, CaCl_2_, KH_2_PO_4_) on In Vitro Shoot Growth of Blackberry, Blueberry, and Saskatoon

**DOI:** 10.3390/plants9080935

**Published:** 2020-07-24

**Authors:** Júlia Hunková, Alena Gajdošová, Monika Szabóová

**Affiliations:** Institute of Plant Genetics and Biotechnology, Plant Science and Biodiversity Center, Slovak Academy of Sciences, 950 07 Nitra, Slovakia; alena.gajdosova@savba.sk (A.G.); m.szaboova@savba.sk (M.S.)

**Keywords:** mesos, mineral nutrition, shoot multiplication, *Rubus fruticosus*, *Vaccinium corymbosum*, *Amelanchier alnifolia*

## Abstract

Berry fruit species are, in many countries, considered biologically and economically valuable and important species of small fruits. The aim of this work was to examine the influence of either decreased or increased mesos concentrations (MgSO_4_, CaCl_2_, and KH_2_PO_4_) on shoot multiplication of five cultivars of three small fruit species (*Amelanchier alnifolia* var. *cusickii*, *Rubus fruticosus* ‘Black Satin’ and ‘Loch Ness’, and *Vaccinium corymbosum* ‘Brigitta Blue’ and ‘Toro’). Mesos nutrients were manipulated from half to four times their base concentration. The results indicate that mesos manipulation significantly influences the number and length of shoots in most of the studied cultivars. The greatest multiplication rate for *A. alnifolia* was achieved with tripled mesos, whereas ‘Black Satin’ and ‘Loch Ness’ reacted positively to a lower (1–2x) concentration of mesos. Decreasing the concentration of mesos to half led to worse quality in both blackberry and Saskatoon shoots. ‘Brigitta Blue’ was more sensitive to greater mesos concentrations compared to ‘Toro’. Optimizing the mineral nutrition of plants cultivated in vitro enhances their multiplication rate and contributes to a higher production of good quality plantlets.

## 1. Introduction

Small fruits, also known as berry crops, are small to moderate-sized fruits produced on perennial herbs, vines, or shrubs. Brambles (blackberry, raspberry and their hybrids), *Ribes* (currant and gooseberry), strawberries, table and winegrapes (*Vitis* spp.), and *Vaccinium* species (blueberry, cranberry, lingonberry, and others) are among the important small fruit crops worldwide [1,2]. Other small fruit species cultivated regionally include *Amelanchier* (serviceberry or Saskatoon), *Sambucus* (elderberries), and *Viburnum* (highbush cranberry or American cranberry bush). The importance of small fruits in horticulture lies in their dual role in the landscape and as food. The fruits themselves are highly prized for their varying shapes, textures, flavors, and colors [1]. Nutritious small fruits are a major human dietary source of phytochemicals including flavonoids and other phenolic compounds, cyanogenic glucosides, phytoestrogens [3], and phenols that are potentially health-promoting and are thought to fight against diseases [4].

In vitro propagation, or micropropagation, has been attractive to researchers for its incredible potential for mass production of a selected genotype in a short time all year-round [5]. The mineral composition of plant tissue culture media is, alongside growth regulators, presumed to play a primarily supportive role in the regeneration process. Nutrient requirements vary among different plant species, but, often, one medium type used throughout cultivation may not be optimal for all stages of explant development [6,7]. Usually, most small fruit species such as strawberries [8,9], raspberries, and blackberries [10,11], along with non-traditional Saskatoon or honeysuckle species [12,13], are cultivated on Murashige and Skoog (MS) medium [14]. However, this medium was originally designed for tobacco calli cultures, not for shoot cultures [15]. Several authors reported that plants cultivated on MS medium display hyperhydricity [16], leaf chlorosis [17], or necrosis [18]. The number of studies focused on culture media mineral composition is rising [19,20,21,22]. Yet, reports focused on media other than MS medium are scant. Some authors [7] manipulated the basal salt composition of modified Gamborg’s B5 medium [23]; the others [24] focused on the composition of Driver and Kuniyuki [25] medium. Kovalchuk et al. [26] focused on modulation of mesos, mineral, and minor nutrients in McCown Woody Plant (WPM) medium [27].

Mesos salts (CaCl_2_·2H_2_O, MgSO_4_·7H_2_O, and KH_2_PO_4_) are currently some of the most studied factors influencing growth of the plants in vitro. Rarely, K_2_SO_4_ is also included in the mesos group [26]. The majority of studies showed that increased mesos salts have a positive effect on growth parameters such as shoot length or shoot number, thereby contributing to a better quality of treated shoots [15,28]. Only a small segment of this research area is oriented toward small fruits, mostly on raspberries (*Rubus ideus* L.) [29,30,31] and nothing is known about the response of other berry species to different mesos contents in culture media. The primary objective of this study was to determine the optimal concentrations of mesos salts for efficient shoot growth of several small fruit cultivars with increased and decreased mesos concentrations being taken into account. Completing the optimizing of growth medium for blackberry, blueberry, and Saskatoon could provide better commercial production.

## 2. Results

### Effect of Cultivar, Mesos Concentration, and Subcultivation on Shoot Number and Length in Studied Cultivars

Analyses of variance proved a significant and highly significant effect (*p* < 0.01) of most of the tested factors (cultivar, mesos concentration, subcultivation) and their interactions on the shoot number and length in each species studied (Table 1 and Table 2).

For *Rubus* cultivars, the average number of shoots after the second subculture was significantly higher compared to the first subculture (‘Loch Ness’—2.78 vs. 2.07, ‘Black Satin’—4.49 vs. 2.89). The same outcome was valid also for *Vaccinium* cultivar ‘Toro’ (6.19 vs. 4.83), but not for ‘Brigitta Blue’. However, the marked increases in shoot number of cv. ‘Black Satin’ and ‘Toro’ were connected to a significant decrease in their shoot length (1.51–1.23 and 1.15–1.04, respectively). No significant differences were observed in the shoot number and length in *A. alnifolia* after two subcultures (Table 3).

**Figure 1:**
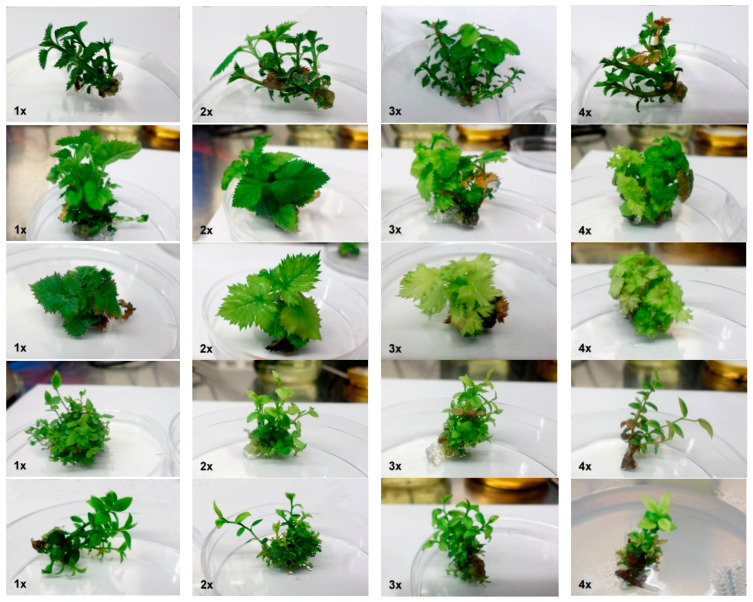
From the first row up to the last row down: *A. alnifolia* var. *cusickii*, *R. fruticosus* cultivars ‘Black Satin’and ‘Loch Ness’, and *V. corymbosum* cultivars ‘Brigitta Blue’and ‘Toro’ after 4 weeks of cultivation on Murashige and Skoog (MS) or McCown Woody Plant (WPM) media with modified mesos concentrations. The numbers indicate the concentration of mesos when 1x mesos = control. The diameter of Petri dishes was six cm.

In *A. alnifolia*, the 3x mesos concentration was significantly the most efficient considering the shoot number and length, whereas the treatment with 4x mesos resulted in the lowest number of shoots (Table 4). Visually, the shoots from 1–3x mesos treatments were healthy with green leaves, but 0.5x mesos treatment often led to shoot-tip drying, and 4x mesos resulted in occasional chlorotic leaves and necrotic shoots (Figure 1).

In *R. fruticosus* cultivars, no mesos treatment differed significantly from the control variants regarding shoot number (Table 4). The best results in ‘Black Satin’ were obtained using a triple concentration of mesos (4.55 shoots/explant). Conversely, this concentration also led to a significant decrease in shoot length. In ‘Loch Ness’ the positive effect of increased mesos concentration on shoot length was observed. The ‘Black Satin’ shoots from variants with 0.5–2x mesos were green, but higher mesos concentrations led to chlorotic, discolored, or necrotic leaves. In ‘Loch Ness’, only the control variant produced vital green shoots (Figure 1). Given the above findings, *R. fruticosus* cultivars reacted positively to 1–2x concentrations of mesos. In *V. corymbosum* ‘Brigitta Blue’, only the highest mesos concentration (4x) had a significantly negative effect on shoot multiplication, and this decrease was related to a significant increase in its shoot length (Table 4). The shoot multiplication in ‘Toro’ did not change significantly under any treatment, suggesting that this cultivar responds uniformly to a wide range of mesos concentrations in culture media. All shoots of blueberry cultivars from 1–3x mesos treatments were healthy and green, but the shoots from 4x mesos treatment displayed chlorotic or discolored leaves (Figure 1).

## 3. Discussion

Little is known about the cultivation of small fruits on nutritional media with customized mineral composition. Analyses of variance in our study confirmed a highly significant effect of most of the tested factors and their interactions on the shoot number and length in studied species. In *A. alnifolia,* only the single effect of subcultivation factor was nonsignificant for shoot number variation, whereas its interaction with the mesos factor was highly significant. In *R. fruticosus,* the explants were affected by all factors except mesos; in *V. corymbosum*, the effects of the cultivar × subcultivation interaction and interaction of all factors were nonsignificant. Previous studies focusing on *R. idaeus* [30,31] showed that mild mesos concentration increases (up to 1.5x) improved the shoot quality and multiplication of all examined cultivars. However, a more detailed subsequent study revealed that the role of each mesos component differed for individual cultivars. In raspberries, increased CaCl_2_ predominantly affected the length of shoots [15]. A similar effect was observed in our study for cultivar ‘Brigitta Blue’ after applying the highest concentrations of mesos in the culture media. Since calcium is a regulator of cell wall growth [28], it may significantly better explain shoot growth in some cultivars. Wada et al. [32], in a mineral analysis of tissues from both treated and control shoots of *Pyrus* spp., also confirmed that the level of calcium increased rapidly in shoots subjected to elevated mesos treatments.

In currently available studies, decreasing the concentration of mesos was rarely beneficial. Poothong and Reed [15] showed that *R. idaeus* ‘Willamette’ grown under low (0.5x) MgSO_4_ concentration developed a higher number of shoots relative to higher (2–3x) concentrations. We found a similar response in blackberry cultivar ’Black Satin’. However, that was the only species–variety combination with this response. Both *A. alnifolia* var. *cusickii* and ‘Loch Ness’ had lower quality shoots when subjected to a decreased mesos concentration. Like Poothong and Reed [15], we observed individualized cultivar responses in both multiplication rate and phenotypic appearance after treatment with different mesos concentrations. *R. fruticosus* cultivar ‘Loch Ness’ and *V. corymbosum* cultivar ‘Brigitta Blue’ were more sensitive to the mesos modulation than *R. fruticosus* cultivar ‘Black Satin’ or *V. corymbosum* cultivar ‘Toro’. Mild mesos increases also reduce the presence of leaf spots and necrosis, as shown in different pear and raspberry cultivars [18,30]. We observed a similar tendency in almost all cultivars after doubling the mesos concentration. 

Another observation in our study was the increased prevalence of chlorosis, especially in both blueberry cultivars and ‘Loch Ness’, after application of the highest mesos concentration. Wada et al. [32] implicated that an elevated phosphate concentration in the media can act as an inhibitor of iron uptake. 

In conclusion, this study presents the effects of manipulating mesos salts in culture media on five cultivars of three different small fruit species. We recommend using triple the mesos concentration to improve the growth of *A. alnifolia* var. *cusickii*. For *R. fruticosus* cultivar ‘Black Satin’, doubling the mesos is preferable. *R. fruticosus* cultivar ‘Loch Ness’ was the most sensitive cultivar for which only the base mesos concentration is recommended. For blueberries, using the base or double mesos concentration for their cultivation is recommended. Very high concentrations of mesos should be avoided for all cultivars. Additional research could provide more detailed insight into the topic of mesos manipulation, considering different cultivars to find optimal conditions for successful micropropagation of small fruit species.

## 4. Materials and Methods 

### 4.1. Plant Material 

As a plant material for all experiments in vitro shoots of *Amelanchier alnifolia* var. *cusickii*, *Rubus fruticosus* L. (cv. ‘Black Satin’ and ‘Loch Ness’), and *Vaccinium corymbosum* L. (cv. ‘Brigitta Blue’ and ‘Toro’) were used.

### 4.2. Shoot Initiation and Multiplication

Sprouts bearing several axillary or apical buds were taken from stock plants, cut into 1–1.5 cm segments, and sterilized with 70% (v/v) ethanol for 2 min followed by immersion in 0.1% (w/v) HgCl_2_ with Tween for 5 min and three rinses in sterile distilled water. Single-node explants were placed vertically in sterile Petri dishes (6 cm in diameter) and filled with the culture medium. In vitro cultures of *Amelanchier* and *Rubus* cultivars were established at MS medium [14], whereas *Vaccinium* cultures were initiated on WPM medium [26]. All media contained 30 g/L sucrose (Slavus, Bratislava, Slovakia) and 8 g/L plant agar (Duchefa Biochemie, Haarlem, The Netherlands) with pH adjusted to 4.4 (WPM medium) or 5.6 (MS medium) before autoclaving 20 min at 1 kg cm^3^ and 121°C. Growth regulators for shoot initiation were used as follows: 1 mg/L 6-benzylaminopurine (BAP) and 0.5 mg/L indole-3-butyric acid (IBA) for *A. alnifolia*; 2 mg/L BAP and 0.2 mg/L IBA for *R. fruticosus*; and 2 mg/L zeatin (ZEA) with 0.2 mg/L indole-3-acetic acid (IAA) for *V. corymbosum*. After six weeks, 10 shoots from each cultivar were transferred to Combiness vessels (Microbox Combiness, Nevele, Belgium) containing multiplication medium. Growth regulators for shoot multiplication were the same as in the induction medium with the exception of *R. fruticosus* (1 mg/L BAP, 0.5 mg/L IBA, and 0.1 mg/L gibberellic acid GA_3_). All plant growth regulators were filter-sterilized before being added to the culture medium. The concentrations of plant growth regulators used follow the earlier published protocols [33,34,35]. Proliferating shoots were subcultured in 4-week intervals. All cultures were maintained in a growth chamber at 22 ± 2°C with a 16 h light period using cool white fluorescent light at a photosynthetic photon flux density 50 μM m^−2^ s^−1^.

### 4.3. Experimental Design

Three mesos macroelements were examined: CaCl_2_·2H_2_O, MgSO_4_·7H_2_O, and KH_2_PO_4_ with concentrations based on standard MS amount (1x (control), 0.5x, 2x, 3x and 4x) and with concentrations based on standard WPM amount (1x (control), 2x, 3x and 4x). 

All mesos components were manipulated simultaneously, and the individual effects of CaCl_2_·2H_2_O, MgSO_4_·7H_2_O, and KH_2_PO_4_ were not examined. Each mesos treatment consisted of 30 explants (six explants/vessel). Two replications per cultivar (60 explants/cultivars) were used for each treatment. The number and length of axillary shoots were evaluated once every four weeks. Thirty randomly chosen explants were selected per each mesos treatment and defined shoots with the height 0.5 cm and above were calculated and measured. The length was measured from the cut on the base of the shoot to the shoot tip with a ruler. The duration of the experiment extended over eight weeks (with two subcultures of the shoots on the fresh culture medium in 4-week intervals). The influence of mesos concentration, subcultivation, and cultivar (except of A. *alnifolia* where only one cultivar was involved in the experiment) on shoot number and length was evaluated.

### 4.4. Statistical Analysis

Obtained data on shoot number and length were evaluated separately in individual species by factorial analysis of variance (two-way ANOVA and three-way ANOVA). Differences among means of individual factor levels were evaluated by multiple-range Duncan’s tests at *p* ≤ 0.05. Software system STATISTICA version 10 (StatSoft, Inc., Tulsa, Oklahoma, USA, 2011) was used for evaluation of all the data obtained.

## Figures and Tables

**Table 1:** Two-way ANOVA test for shoot number in *Amelanchier alnifolia* var. *cusickii* and three-way ANOVA test for shoot number in *Rubus fruticosus* and *Vaccinium corymbosum* cultivars.

Effect	*A. alnifolia*			*R. fruticosus*			*V. corymbosum*		
	df	F	*p*	df	F	*p*	df	F	*p*
cultivar		-		1	44.0736	0.000000	1	58.484	0.000000
mesos	4	5.1395	0.000523	4	2.1595	0.072593	3	13.000	0.000000
subcultivation	1	0.0621	0.803403	1	36.4831	0.000000	1	7.919	0.005107
cultivar × mesos		-		4	3.3075	0.010924	3	9.332	0.000005
cultivar × subcult.		-		1	7.2468	0.007360	1	0.994	0.319241
mesos × subcultivation	4	7.7551	0.000006	4	2.9868	0.018731	3	12.761	0.000000
cultivar × mesos×subcultivation		-		4	4.5503	0.001296	3	10.636	0.000001
Error	275			464			447		

Note: df—degrees of freedom; F—F-value; *p*—probability value, subcultivation—four weeks of cultivation.

**Table 2:** Two-way ANOVA test for shoot length in *A. alnifolia* var. *cusickii* and three-way ANOVA test for shoot length in *R. fruticosus* and *V. corymbosum* cultivars.

Effect	*A. alnifolia*			*R. fruticosus*			*V. corymbosum*		
	df	F	*p*	df	F	*p*	df	F	*p*
cultivar		-		1	221.390	0.000000	1	27.38	0.000000
mesos	4	24.374	0.000000	4	10.415	0.000000	3	28.36	0.000000
subcultivation	1	19.817	0.000009	1	20.028	0.000008	1	6.40	0.011443
cultivar × mesos		-		4	18.556	0.000000	3	10.14	0.000001
cultivar × subcult.		-		1	20.815	0.000005	1	10.27	0.001363
mesos × subcult.	4	17.862	0.000000	4	9.399	0.000000	3	18.98	0.000000
cultivar × mesos×subcultivation		-		4	4.468	0.001363	3	1.14	0.329475
Error	1913			1525			3146		

Note: df—degrees of freedom; F—F-value; *p*—probability value, subcultivation—four weeks of cultivation.

**Table 3:** The effect of cultivar and subcultivation on shoot number and length in *R. fruticosus*, *V. corymbosum and A. alnifolia* var. *cusickii*.

Species	Cultivar	Subcultivation	Number of Shoots (±SD)	Length of Shoots in cm (±SD)
			Mean	Mean
*R. fruticosus*	‘Loch Ness’	1	2.07 ± 1.17c	0.93 ± 0.49c
		2	2.78 ± 2.12b	0.98 ± 0.51c
	‘Black Satin’	1	2.89 ± 2.06b	1.51 ± 0.59a
		2	4.49 ± 2.75a	1.23 ± 0.48b
*V. corymbosum*	‘Toro’	1	4.83 ± 2.46c	1.15 ± 0.65a
		2	6.19 ± 2.93b	1.04 ± 0.52b
	‘Brigitta Blue’	1	7.74 ± 5.40a	0.94 ± 0.57c
		2	8.53 ± 5.62a	0.99 ± 0.51bc
*A. alnifolia* var. *cusickii*		1	6.60 ± 4.91a	1.62 ± 0.70a
		2	6.90 ± 5.36a	1.68 ± 0.68a

Mean values within each species followed by the same letter were not significantly different at *p* ≤ 0.05 according to the Duncan test. Note: subcultivation 1—first four weeks of cultivation, subcultivation 2—next four weeks of cultivation.

**Table 4:** The effect of different mesos concentrations on number and length of shoots in *A. alnifolia* var. *cusickii*, *R. fruticosus*, and *V. corymbosum*.

Species	Cultivar	Mesos Concentration	Mean Number of Shoots (±SD)	Mean Length of Shoots (±SD)
*A. alnifolia* var. *cusickii*		0.5×	7.66 ± 4.56ab	1.61 ± 0.61b
		1×	6.07 ± 5.37bc	1.59 ± 0.64b
		2×	6.10 ± 3.96bc	1.47 ± 0.64c
		3×	8.64 ± 5.77a	1.84 ± 0.74a
		4×	5.02 ± 5.22c	1.69 ± 0.75b
*R. fruticosus*	‘Black Satin’	0.5×	3.02 ± 1.58bc	1.41 ± 0.58a
		1×	3.77 ± 2.22ab	1.35 ± 0.59a
		2×	3.93 ± 2.48ab	1.31 ± 0.52ab
		3×	4.55 ± 3.65a	1.21 ± 0.40bc
		4×	3.34 ± 2.40b	1.43 ± 0.61a
*R. fruticosus*	‘Loch Ness’	0.5×	2.26 ± 1.34c	0.62 ± 0.29f
		1×	2.32 ± 1.58c	0.83 ± 0.42e
		2×	2.30 ± 2.20c	1.14 ± 0.54c
		3×	2.41 ± 1.40c	0.96 ± 0.47d
		4×	3.04 ± 2.24bc	1.18 ± 0.50c
*V. corymbosum*	‘Brigitta Blue’	1×	9.32 ± 5.90ab	0.93 ± 0.45c
		2×	10.07 ± 5.39a	1.01 ± 0.48bc
		3×	8.43 ± 5.81b	0.82 ± 0.47d
		4×	4.81 ± 3.06c	1.20 ± 0.76a
*V. corymbosum*	‘Toro’	1×	5.61 ± 2.65c	1.14 ± 0.56a
		2×	5.54 ± 2.65c	1.00 ± 0.54bc
		3×	5.82 ± 3.11c	1.04 ± 0.53b
		4×	5.17 ± 2.74c	1.18 ± 0.66a

Mean values within each species followed by the same letter were not significantly different at *p* ≤ 0.05 according to the Duncan test.
